# Sorafenib fails to trigger ferroptosis across a wide range of cancer cell lines

**DOI:** 10.1038/s41419-021-03998-w

**Published:** 2021-07-13

**Authors:** Jiashuo Zheng, Mami Sato, Eikan Mishima, Hideyo Sato, Bettina Proneth, Marcus Conrad

**Affiliations:** 1 Helmholtz Zentrum München, Institute of Metabolism and Cell Death, Ingolstädter Landstr. 1, 85764 Neuherberg, Germany; 2 Laboratory of Biochemistry and Molecular Biology, Department of Medical Technology, Faculty of Medicine, Niigata University, Niigata, Japan; 3 Sakeology Center, Niigata University, 8050 Ikarashi 2-no-cho, Nishi-ku Niigata, 950-2181 Japan; 4 Pirogov National Research Medical University, Laboratory of Experimental Oncology, Ostrovityanova 1, Moscow, 117997 Russia

**Keywords:** Cell death, Small molecules

## Abstract

Sorafenib, a protein kinase inhibitor approved for the treatment of hepatocellular carcinoma and advanced renal cell carcinoma, has been repeatedly reported to induce ferroptosis by possibly involving inhibition of the cystine/glutamate antiporter, known as system x_c_^−^. Using a combination of well-defined genetically engineered tumor cell lines and canonical small molecule ferroptosis inhibitors, we now provide unequivocal evidence that sorafenib does not induce ferroptosis in a series of tumor cell lines unlike the cognate system x_c_^−^ inhibitors sulfasalazine and erastin. We further show that only a subset of tumor cells dies by ferroptosis upon sulfasalazine and erastin treatment, implying that certain cell lines appear to be resistant to system x_c_^−^ inhibition, while others undergo ferroptosis-independent cell death. From these findings, we conclude that sorafenib does not qualify as a *bona fide* ferroptosis inducer and that ferroptosis induced by system x_c_^−^ inhibitors can only be achieved in a fraction of tumor cell lines despite robust expression of SLC7A11, the substrate-specific subunit of system x_c_^−^.

## Introduction

**Fig. 1:**
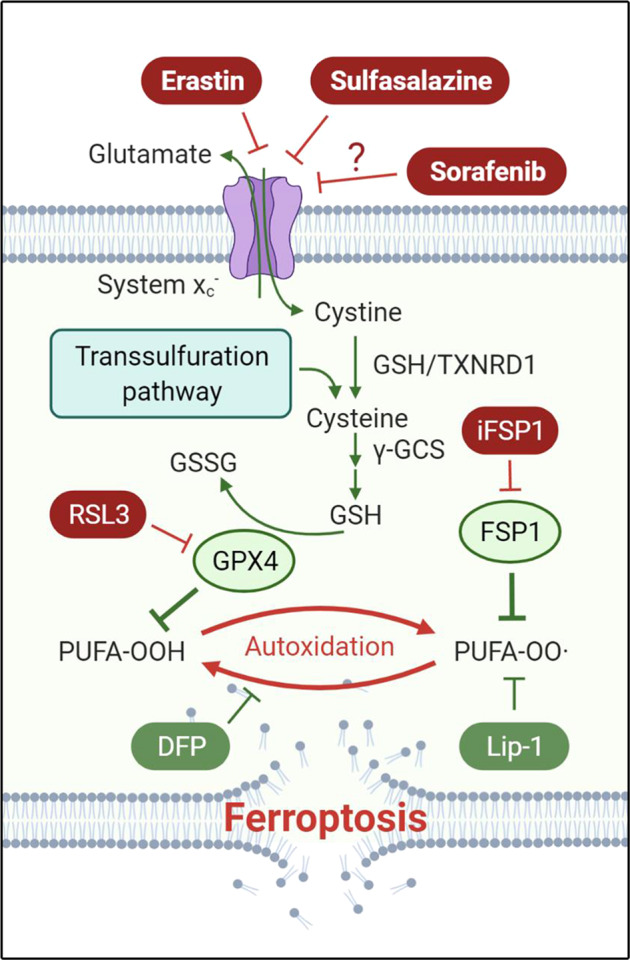
Schematic representation of the key mechanisms involved in ferroptosis control. Cell death via ferroptosis is executed by iron-dependent lipid peroxidation occurring on polyunsaturated fatty acid (PUFA)-enriched phospholipids. This process can be prevented by iron chelators like deferiprone (DFP) and radical-trapping antioxidants like liproxstatin-1 (Lip-1). Glutathione peroxidase 4 (GPX4) and ferroptosis suppressor protein 1 (FSP1) are the main systems to counteract lipid peroxidation and ferroptosis. Inhibition of GPX4 by *(1S,3R)*-RSL3 (RSL3) and FSP1 by iFSP1, respectively, sensitizes cells to ferroptosis. Full activity of GPX4 requires sufficient glutathione (GSH/GSSG), whereby the synthesis of GSH relies on its rate-limiting substrate cysteine and the enzymatic machinery including γ-glutamylcysteine synthetase (γ-GCS). Endogenous cysteine, though it might be partially provided through the transsulfuration pathway, is largely generated via GSH- or thioredoxin reductase 1 (TXNRD1)-mediated reduction of cystine, with cystine being mainly imported from the extracellular space by the cystine/glutamate antiporter system x_c_^−^, at least in cultured cells. While erastin and sulfasalazine are classic ferroptosis inducers by inhibiting system x_c_^−^, it has remained obscure whether sorafenib is a *bona fide* system x_c_^−^ inhibitor or ferroptosis inducer.

Ferroptosis is a unique type of cell death characterized by an overwhelming lipid peroxidation downstream of metabolic dysfunctions [1]. The term “ferroptosis” was first coined in 2012 through the detailed characterization of the lethal mechanisms of erastin and *(1S,3R)*-RSL3 (RSL3), both of which trigger this iron-dependent cell death modality [2]. Erastin irreversibly targets system x_c_^−^ [3], an amino acid antiporter composed of the substrate-specific light chain SLC7A11 (alias xCT) and the heavy chain SLC3A2 (alias 4F2), that works by exchanging extracellular cystine (i.e., the oxidized dimeric form of cysteine) for intracellular glutamate at a 1:1 ratio (Fig. 1) [4–6]. RSL3 irreversibly inhibits the phospholipid hydroperoxidase glutathione peroxidase 4 (GPX4) [7, 8], a selenoenzyme that functions by reducing hydroperoxide groups in polyunsaturated fatty acid residues esterified in phospholipids to their corresponding alcohols [9]. System x_c_^−^ acts upstream of GPX4, because cystine imported by system x_c_^−^ is reduced to cysteine and then used for the synthesis of glutathione (GSH), the main electron donor for GPX4 (Fig. 1). Moreover, the reductive microenvironment maintained by system x_c_^−^ is required for cellular selenium uptake and thus the biosynthesis of GPX4 [10, 11]. Previous data also suggest that forced expression of xCT is sufficient to protect cells against oxidative stress by maintaining a cystine/cysteine redox cycle over the cell membrane [12, 13]. In addition to system x_c_^−^, the transsulfuration pathway may provide cysteine to some extent [14]. Besides the cyst(e)ine/GSH/GPX4 axis, ferroptosis suppressor protein 1 (FSP1) was recently identified to counteract ferroptosis in a GSH- and GPX4-independent manner [15, 16]. Unlike GPX4, FSP1 functions by reducing ubiquinone yielding ubiquinol, which in turn can directly, or indirectly by recycling α-tocopherol, act as radical-trapping antioxidant (RTA) to scavenge lipid radicals in membranes, thereby halting lipid peroxidation and associated ferroptosis.

Since the coining of ferroptosis, inhibition of system x_c_^−^ has been appreciated as a classic way to induce ferroptosis. Among the known system x_c_^−^ inhibitors, erastin is, without doubt, the most widely used. A number of cell lines including diffuse large B cell lymphoma SU-DHL-8 and WSU-DLCL-2, renal cell carcinoma Caki-1 and 786-O [7], lung cancer Calu-1, and fibrosarcoma HT1080 [2] have been demonstrated to be sensitive to erastin-induced ferroptosis. Nevertheless, erastin has limited solubility and low metabolic stability precluding its applicability in vivo [7]. Sulfasalazine is a clinically approved drug that has long been used for the treatment of rheumatoid arthritis and inflammatory bowel disease. In 2001, sulfasalazine was recognized to be a system x_c_^−^ inhibitor through drug screening [17]. Regardless of the much lower potency compared to erastin, sulfasalazine is an independent compound for system x_c_^−^ inhibition that has been widely acknowledged largely before erastin was introduced [2, 18]. Sorafenib is a multikinase inhibitor used in the therapy of advanced kidney and liver cancer. It was first proposed to act as a ferroptosis inducer in 2013, based on the evidence that the cytotoxic effect of sorafenib on hepatoma Huh7 cells was significantly prevented by the iron chelator deferoxamine and the RTA ferrostatin-1 [19]. Subsequently, sorafenib was found to induce ferroptosis via system x_c_^−^ inhibition in HT1080 cells as β-mercaptoethanol (β-ME), deferoxamine, and ferrostatin-1 all rescued sorafenib-induced cell death to some extent [3]. In addition, sorafenib has been suggested to induce ferroptosis in kidney adenocarcinoma ACHN [20] and hepatoma HepG2 [21] cells. Albeit these numerous reports, additional experimental evidence suggested that sorafenib has low selectivity and therefore may trigger other lethal mechanisms [3]. As such, in the present study, we sought to explore whether sorafenib indeed qualifies as a robust *bona fide* system x_c_^−^ inhibitor or ferroptosis inducer in a wide range of cell lines.

## Materials and methods

### Reagents

Reagents used in this work include sorafenib (sc-220125, Santa Cruz), sulfasalazine (S0883, VWR), erastin (329600, Sigma-Aldrich), β-ME (31350010, ThermoFisher), liproxstatin-1 (Lip-1) (S7699, Selleck Chemicals), deferiprone (DFP) (379409, Sigma-Aldrich), *(1S,3R)*-RSL3 (RSL3) (Cay19288, Cayman Chemical), iFSP1 (8009-2626, ChemDiv) [15], and [^14^C]cystine (NEC854010UC, PerkinElmer).

### Cell lines

The human fibrosarcoma HT1080, melanoma A375, lung cancer A549, colon cancer HT29, breast cancer MDA-MB-436, glioma U-373, kidney cancer UMRC2, and various hepatoma HLE, HLF, HepG2, and Huh7 (HepG2 and Huh7 were kind gifts from Dr. Robert Schneider, Helmholtz Zentrum München) cell lines were purchased from ATCC and cultured according to ATCC guidelines. Human HEK293T cells were purchased from Clontech. The mouse melanoma cell line B16F10 was obtained from Cell Bank, RIKEN Bio-Resource Research Center (Ibaraki, Japan), and *Slc7a11*^*KO*^ and *Slc7a11*^*OE*^ B16F10 cells were established as reported previously [22]. *SLC7A11*^*KO*^ HT1080 and HEK293T cells were generated from the parental cell lines, and *SLC7A11*^*OE*^ cells were established using *SLC7A11*^*KO*^ cells following the methods described below.

### Generation of *SLC7A11*^*KO*^ and *SLC7A11*^*OE*^ HT1080 and HEK293T cells

*SLC7A11*^*KO*^ cells were generated using CRISPR/Cas9 system-based technology. In brief, two single guide RNAs targeting *SLC7A11* exons 1 and 3, respectively, were designed using the platform https://www.vbc-score.org/ [23]. The two guides were then cloned in the *BsmB*I-digested lentiCRISPR v2 blast and lentiCRISPR v2 puro vectors (Addgene), respectively. The vectors were co-lipofected into HT1080 or HEK293T cells using the X-tremeGene HP DNA Transfection Reagent (Sigma-Aldrich) in the presence of 50 μM β-ME. After 24 h incubation, transfected cells were trypsinized and selected with both puromycin (2 μg/ml) and blasticidin (10 μg/ml) in 10-cm dishes for 96 h in the presence of 50 μM β-ME. Cells that survived the selection were collected and 100 cells were plated on three 96-well plates to allow the formation of single-cell clones. *SLC7A11*^*KO*^ single-cell clones were verified first by β-ME removal (*SLC7A11*^*KO*^ cells would die in the absence of β-ME) followed by immunoblotting.

To generate *SLC7A11*^*OE*^ cells, *SLC7A11*^*KO*^ cells were lipofected with the human xCT expression vector pCAG-3SIP-hxCT and then selected in β-ME-free medium 24 h later for one week. Cells that survived the selection were collected and single-cell clones were obtained as described above. *SLC7A11*^*OE*^ cells were confirmed by immunoblotting.

### Measurement of cystine transport activity

Uptake of cystine in WT and *SLC7A11*^*OE*^ HT1080 cells was measured as described [6]. Briefly, 2 × 10^5^ cells were plated on 35 mm dishes 24 h before the experiment. Cells were washed three times in pre-warmed PBS(+) G (10 mM phosphate-buffered saline pH 7.4, containing 0.01% CaCl_2_, 0.01% MgCl_2_ · 6H_2_O, and 0.1% glucose) and then incubated in 0.5 ml of pre-warmed uptake medium at 37 °C for 2 min. The uptake medium contained 50 μM cystine plus [^14^C]cystine (0.1 μCi/ml) and 10 µM erastin or sorafenib as indicated. Uptake was terminated by rapidly rinsing the cells three times with ice-cold PBS. The cells were then lysed by 500 µl of 0.5 N NaOH and the lysate was used for radioactivity measurement by the liquid scintillation counter (LSC-5100, ALOKA, Japan).

### Immunoblotting

Cells were lysed in LCW lysis buffer (0.5% Triton-X-100, 0.5% sodium deoxycholate salt, 150 mM NaCl, 20 mM Tris-HCl, 10 mM EDTA, 30 mM Na-pyrophosphate) containing protease and phosphatase inhibitor mixture (both Roche, Mannheim, Germany), and cell debris were removed by centrifugation (12,000×*g*, 4 °C, 20 min). Protein concentration was determined by the Pierce BCA Protein Assay kit (ThermoScientific, Bonn, Germany) according to the manufacturer’s protocol. Equal amounts of proteins (25 μg per lane) were separated by 12% SDS-PAGE (Bio-Rad) and blotted onto a PVDF membrane (Bio-Rad). Primary antibodies against human xCT (1:10; Rat IgG2a monoclonal antibody, clone 3A12-1-1-1, developed in-house) [15], GPX4 (1:1000; ab125066, Abcam), FSP1 (1:1000, sc-377120, Santa Cruz), cystathionine-β-synthase (CBS) (1:1000, GTX628777, GeneTex), cystathionine γ-lyase (CSE) (1:1000, GTX113409, GeneTex), glutamate–cysteine ligase catalytic subunit (GCLC) (1:200, sc-22755, Santa Cruz), glutamate–cysteine ligase modifier subunit (GCLM) (1:200, sc-55585, Santa Cruz), and valosin containing protein (VCP) (1:2000; ab11433, Abcam) were used.

### Cell viability

Cells were seeded on 96-well plates until they reached ~50–60% confluency the next day. Cells were then treated with increasing concentrations of ferroptosis modulating compounds (sorafenib, sulfasalazine, erastin, RSL3 in a 7-point dilution), in the absence or presence of 50 μM β-ME, 1 μM Lip-1, 100 μM DFP, or 5 μM iFSP1. Cell viability was assessed 24 h later using AquaBluer as an indicator of viable cells according to the manufacturer’s instructions (MultiTarget Pharmaceuticals, LLC) and as performed previously [8].

### Assessment of lipid peroxidation using C11-BODIPY

WT HT1080 cells were seeded on six-well plates (5 × 10^4^ cells/well) one day prior to the experiment. On the following day, cells were treated with either 10 μM erastin or sorafenib in the absence or presence of 1 μM Lip-1. Four hours later, cells were washed with PBS and incubated with 1.5 μM BODIPY 581/591 C11 (ThermoFisher) for 30 min at 37 °C. Then, cells were trypsinized and resuspended in 300 μl of HBSS (Gibco). The cell suspension was strained through a 40 μm cell strainer (Falcon tube with cell strainer CAP) and analyzed using a flow cytometer (CytoFLEX, Beckman Coulter) with a 488-nm laser paired with a 530/30 nm bandpass filter. At least 10,000 events were analyzed per sample. Data were analyzed using FlowJo Software (Treestar, Inc.).

### Determination of intracellular GSH (reduced form) by HPLC-ECD

WT HT1080, Huh7, and MDA-MB-436 cells were seeded on 10 cm plates until they reached approximately 70-80% confluency the next day. Cells were then treated with 10 μM erastin for 4 h. Afterward, cells were washed with PBS and scraped off with 300 µl of ice-cold PCA solution (0.4 N perchloric acid; 100 nM EDTA). The cells were vortexed for 1 min and centrifuged at 20,000×*g* for 10 min at 4 °C. The cell debris was used for protein quantification with the Pierce BCA Protein Assay Kit (ThermoScientific) as described in the manufacturer’s protocol. The clear supernatant (300 µl) was transferred to a HPLC vial and analyzed using isocratic HPLC-ECD system (Dionex Ultimate 3000) with a Hypersil GOLD™ C18 LC selectivity column (3 μm, 150 × 2.1 mm, ThermoFisher). The mobile phase consisted of 25 mM sodium dihydrogenphosphate, 1.4 mM 1-octanesulfonic acid, and 2% (v/v) acetonitrile, and phosphoric acid was added dropwise to achieve a final pH of 2.65. GSH concentrations are normalized to total protein content.

## Results

### Sorafenib does not induce ferroptosis in human and mouse cancer cell lines

**Fig. 2:**
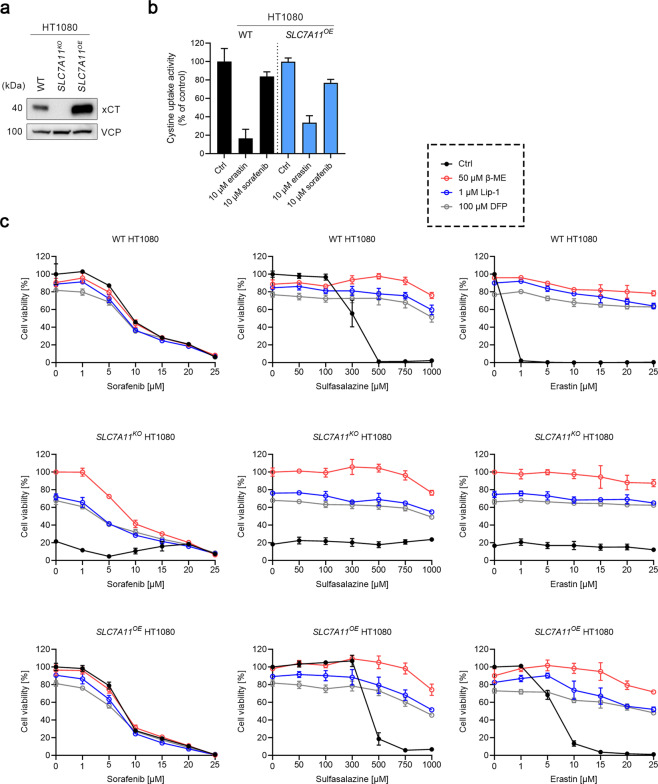
Sorafenib does not induce ferroptosis in HT1080 cells. **a** Immunoblots of xCT in wild-type (WT), *SLC7A11* knockout (*SLC7A11*^*KO*^), and *SLC7A11* overexpressing (*SLC7A11*^*OE*^) HT1080 cells. Valosin-containing protein (VCP) was used as a loading control. **b** Cystine uptake activity of WT and *SLC7A11*^*OE*^ HT1080 cells in the presence of either 10 µM erastin or sorafenib. Data are presented as mean ± s.d. (*n* = 4). **c** Cell viability of WT, *SLC7A11*^*KO*^, and *SLC7A11*^*OE*^ HT1080 cells treated with indicated concentrations of sorafenib, sulfasalazine, or erastin, in the absence or presence of 50 μM β-mercaptoethanol (β-ME), 1 μM Lip-1, or 100 μM DFP for 24 h. Data are presented as mean ± s.d. of *n* = 3 wells of a 96-well plate from one representative of three independent experiments.

**Fig. 3:**
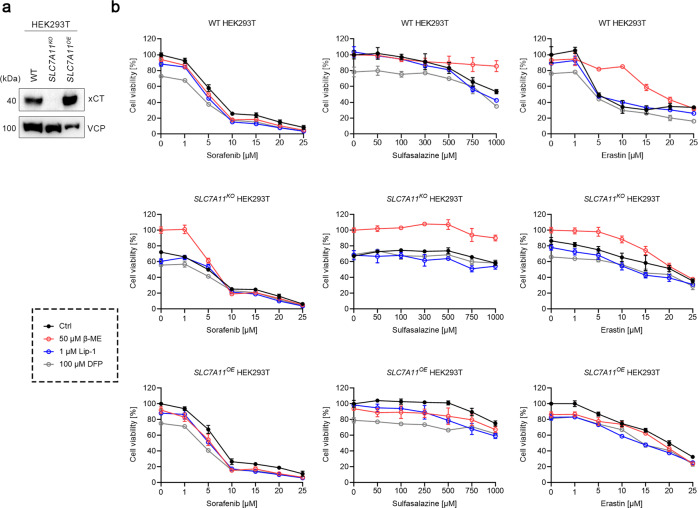
Sorafenib does not induce ferroptosis in HEK293T cells. **a** Immunoblots of xCT in WT, *SLC7A11*^*KO*^, and *SLC7A11*^*OE*^ HEK293T cells. VCP was used as a loading control. **b** Cell viability of WT, *SLC7A11*^*KO*^, and *SLC7A11*^*OE*^ HEK293T cells treated with indicated concentrations of sorafenib, sulfasalazine, or erastin, in the absence or presence of 50 μM β-ME, 1 μM Lip-1, or 100 μM DFP for 24 h. Data are presented as mean ± s.d. of *n* = 3 wells of a 96-well plate from one representative of three independent experiments.

To determine whether sorafenib is a *bona fide* ferroptosis inducer by acting through inhibition of system x_c_^−^, we first genetically engineered expression of SLC7A11 in the human fibrosarcoma cell line HT1080 (a widely used cell line in ferroptosis research), as well as the human embryonic kidney cell line HEK293T. To this end, the gene for the substrate-specific subunit of system x_c_^−^, *SLC7A11*, was disrupted using CRISPR/Cas9 technology (in the following referred to as *SLC7A11*^*KO*^). To rule out clonal effects, SLC7A11 was re-expressed in the *SLC7A11* null background by lipotransfection (in the following referred to as *SLC7A11*^*OE*^). The correct manipulation of the cell lines was verified by immunoblotting (Figs. 2a and 3a).

Next, we measured the cystine uptake activity in WT and *SLC7A11*^*OE*^ HT1080 cells in the presence or absence of either 10 μM erastin or sorafenib. Unlike erastin, which robustly blocked cystine uptake (~85% inhibition) in WT cells and exerted only relatively mild inhibitory effects (~70% inhibition) on *SLC7A11*^*OE*^ cells, sorafenib lowered cystine uptake in both WT and *SLC7A11*^*OE*^ cells only marginally, corroborating that sorafenib is not a *bona fide* xCT inhibitor. Indeed, the slight inhibition of sorafenib might be due to its known cytotoxic effects, since the inhibition could not be reversed by xCT overexpression (Fig. 2b).

Wild-type (WT), *SLC7A11*^*KO*^, and *SLC7A11*^*OE*^ HT1080 cells were then treated with increasing concentrations of sorafenib, sulfasalazine, or erastin, in the presence or absence of 50 μM β-ME, 1 μM Lip-1, or 100 μM DFP (Fig. 2c). Sorafenib exerted similar cytotoxic effects on the three cell lines regardless of the different expression levels of xCT (Fig. 2c, left panels). Furthermore, β-ME, which acts as a shuttle of cystine by forming a mixed disulfide with extracellular cystine and thus enables cells to take up cystine in the absence of xCT [24], and two ferroptosis inhibitors (i.e., Lip-1 and DFP), did not rescue the lethal effects upon sorafenib treatment, albeit Lip-1 did counteract the lipid peroxidation induced by sorafenib (Supplementary Fig. S1). By stark contrast, the cytotoxic effects of sulfasalazine and erastin were significantly mitigated in the presence of β-ME, Lip-1, or DFP (Fig. 2c, middle panels and right panels). Moreover, *SLC7A11*^*OE*^ cells appeared to be more resistant to sulfasalazine and erastin relative to WT cells. These results thus indicate that sorafenib does not trigger ferroptosis in HT1080 cells.

We next asked whether sorafenib induces ferroptosis in HEK293T cells. WT, *SLC7A11*^*KO*^, and *SLC7A11*^*OE*^ HEK293T cells were treated as described in the foregoing (Fig. 3b). As in HT1080 cells, the cytotoxic effect of sorafenib on HEK293T cells was not affected by either the xCT expression level or the presence of β-ME, Lip-1, and DFP, while sulfasalazine and erastin were less potent in *SLC7A11*^*OE*^ cells than in WT parental cells. Interestingly, only β-ME but not Lip-1 or DFP showed a protective effect on WT HEK293T cells upon sulfasalazine or erastin treatment, suggesting that inhibition of xCT does not induce ferroptosis in HEK293T cells. Nevertheless, *SLC7A11*^*KO*^ cells still die in response to β-ME withdrawal (data not shown). Like for HT1080 cells, these results suggest that sorafenib, sulfasalazine, and erastin all failed to induce ferroptosis in HEK293T cells, even though sulfasalazine and erastin did inhibit xCT.

**Fig. 4:**
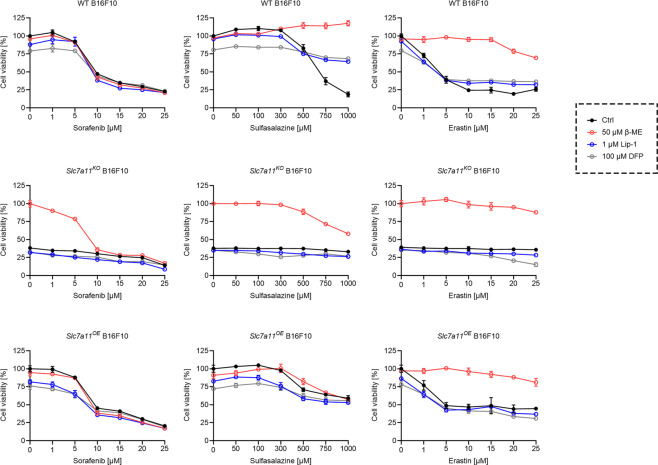
Sorafenib does not induce ferroptosis in B16F10 cells. Cell viability of WT, *Slc7a11*^*KO*^, and *Slc7a11*^*OE*^ B16F10 cells treated with indicated concentrations of sorafenib, sulfasalazine, or erastin, in the absence or presence of 50 μM β-ME, 1 μM Lip-1, or 100 μM DFP for 24 h. Data are presented as mean ± s.d. of *n* = 3 wells of a 96-well plate from one representative of three independent experiments.

We then studied the effects of sorafenib in the highly metastatic murine B16F10 cell line. WT, *SLC7A11*^*KO*^, and *SLC7A11*^*OE*^ B16F10 cells, which were established previously [22], were treated as above (Fig. 4). Again, the cytotoxic effect of sorafenib on B16F10 cells was not affected by xCT expression or the presence of β-ME, Lip-1, or DFP, while sulfasalazine and erastin became less potent upon xCT overexpression and β-ME treatment. Consistent with our previous report [22], *SLC7A11*^*KO*^ cells could only survive in the presence of β-ME, but not in the presence of Lip-1 and DFP. Furthermore, Lip-1 and DFP had no protective effect upon erastin treatment. However, we did observe that Lip-1 and DFP increased the cell viability of WT B16F10 cells when treated with high concentrations (>750 μM) of sulfasalazine. Collectively, these results suggest that sulfasalazine, but not sorafenib or erastin, induced ferroptosis in B16F10 cells, even though sulfasalazine and erastin both inhibited xCT.

### Sorafenib fails to trigger ferroptosis in cell lines with high xCT expression

**Fig. 5:**
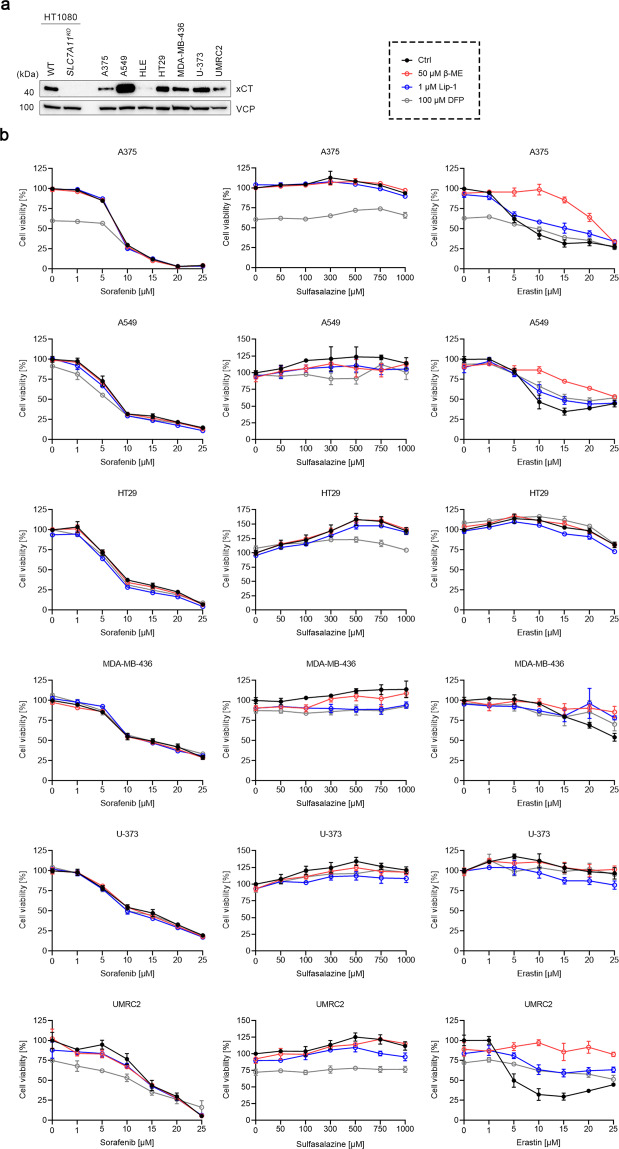
Sorafenib fails to trigger ferroptosis among various cell lines with high xCT expression. **a** Immunoblots of xCT in WT HT1080, *SLC7A11*^*KO*^ HT1080, A375 melanoma, A549 lung cancer, HLE hepatoma, HT29 colon cancer, MDA-MB-436 breast cancer, U-373 glioma, and UMRC2 kidney cancer cells. VCP was used as a loading control. **b** Cell viability of A375, A549, HT29, MDA-MB-436, U-373, and UMRC2 cells treated with indicated concentrations of sorafenib, sulfasalazine, or erastin, in the absence or presence of 50 μM β-ME, 1 μM Lip-1, or 100 μM DFP for 24 h. Data are presented as mean ± s.d. of *n* = 3 wells of a 96-well plate from one representative of three independent experiments.

To further explore whether sorafenib may induce ferroptosis in cancer cell lines known to show robust xCT expression [15], human melanoma A375, lung cancer A549, hepatoma HLE, colon cancer HT29, breast cancer MDA-MB-436, glioma U-373, and kidney cancer UMRC2 cells were selected and the expression level of xCT was determined by immunoblotting (Fig. 5a). Among the seven selected cell lines, the hepatoma HLE showed the lowest xCT expression. Therefore, the six cell lines excluding HLE were treated as outlined above (Fig. 5b). Consistent with our data from the HT1080, HEK293T, and B16F10 cell lines, all six cell lines succumbed to sorafenib treatment in a concentration-dependent manner, which was not affected by the presence of β-ME, Lip-1, or DFP. At the same time, these cell lines appeared to be quite resistant to sulfasalazine. Moreover, three of the six cell lines, namely HT29, MDA-MB-436, and U-373, were even resistant to erastin. Although A375 and A549 cells were sensitive to erastin, only β-ME but not Lip-1 or DFP showed an obvious protective effect, suggesting erastin does not induce robust ferroptosis in these two cell lines. Like in UMRC2 cells, erastin-induced cell death was fully rescued by β-ME and partially rescued by Lip-1 and DFP. These results thus suggest that sorafenib does not trigger ferroptosis in all these six cell lines, and that cells with high xCT expression are not necessarily sensitive to xCT inhibition-induced cell death.

### Sorafenib does not cause ferroptosis in human hepatoma cell lines

**Fig. 6:**
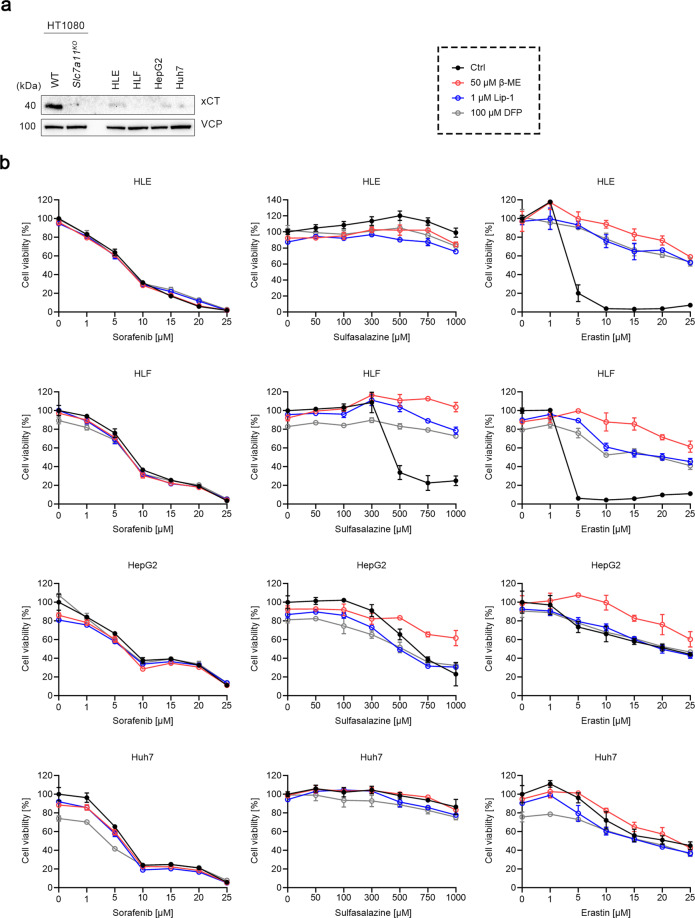
Sorafenib does not cause ferroptosis in human hepatoma cell lines. **a** Immunoblots of xCT in WT HT1080, *SLC7A11*^*KO*^ HT1080, and HLE, HLF, HepG2, Huh7 hepatoma cells. VCP was used as a loading control. **b** Cell viability of HLE, HLF, HepG2, and Huh7 cells treated with indicated concentrations of sorafenib, sulfasalazine, or erastin, in the absence or presence of 50 μM β-ME, 1 μM Lip-1, or 100 μM DFP for 24 h. Data are presented as mean ± s.d. of *n* = 3 wells of a 96-well plate from one representative of three independent experiments.

Given that sorafenib is mainly used for hepatocellular carcinoma treatment and that most of the previous studies showed induction of ferroptosis by sorafenib in hepatoma cell lines, we selected HLE, HLF, HepG2, and Huh7 to examine whether sorafenib elicits ferroptosis in human hepatoma cells. Interestingly, all these four cell lines displayed extremely low xCT expression compared to HT1080 cells (Fig. 6a). Consistent with the other cell lines, sorafenib-induced cell death among these four cell lines was not prevented by the presence of β-ME, Lip-1, or DFP (Fig. 6b, left panels). Sulfasalazine failed to kill HLE and Huh7 cells but induced robust ferroptotic cell death in HLF cells, as indicated by the protective effects of β-ME, Lip-1, and DFP (Fig. 6b, middle panels). HepG2 cells were also sensitive to sulfasalazine, but only β-ME and not Lip-1 or DFP showed a protective effect. Indeed, similar results were observed when HepG2 cells were treated with erastin (Fig. 6b, right panels). By contrast, erastin-induced robust ferroptosis in HLE and HLF cells. As to Huh7 cells, they showed a similar sensitivity to erastin as HepG2 cells, but none of β-ME, Lip-1, or DFP rescued the cells from dying. In accordance with the data above, these findings show that sorafenib fails to cause ferroptosis in hepatoma cells, nor in cells like HLF which are highly sensitive to xCT inhibition-induced ferroptosis.

### Cells resistant to xCT inhibition are sensitive to co-treatment with RSL3 and iFSP1

**Fig. 7:**
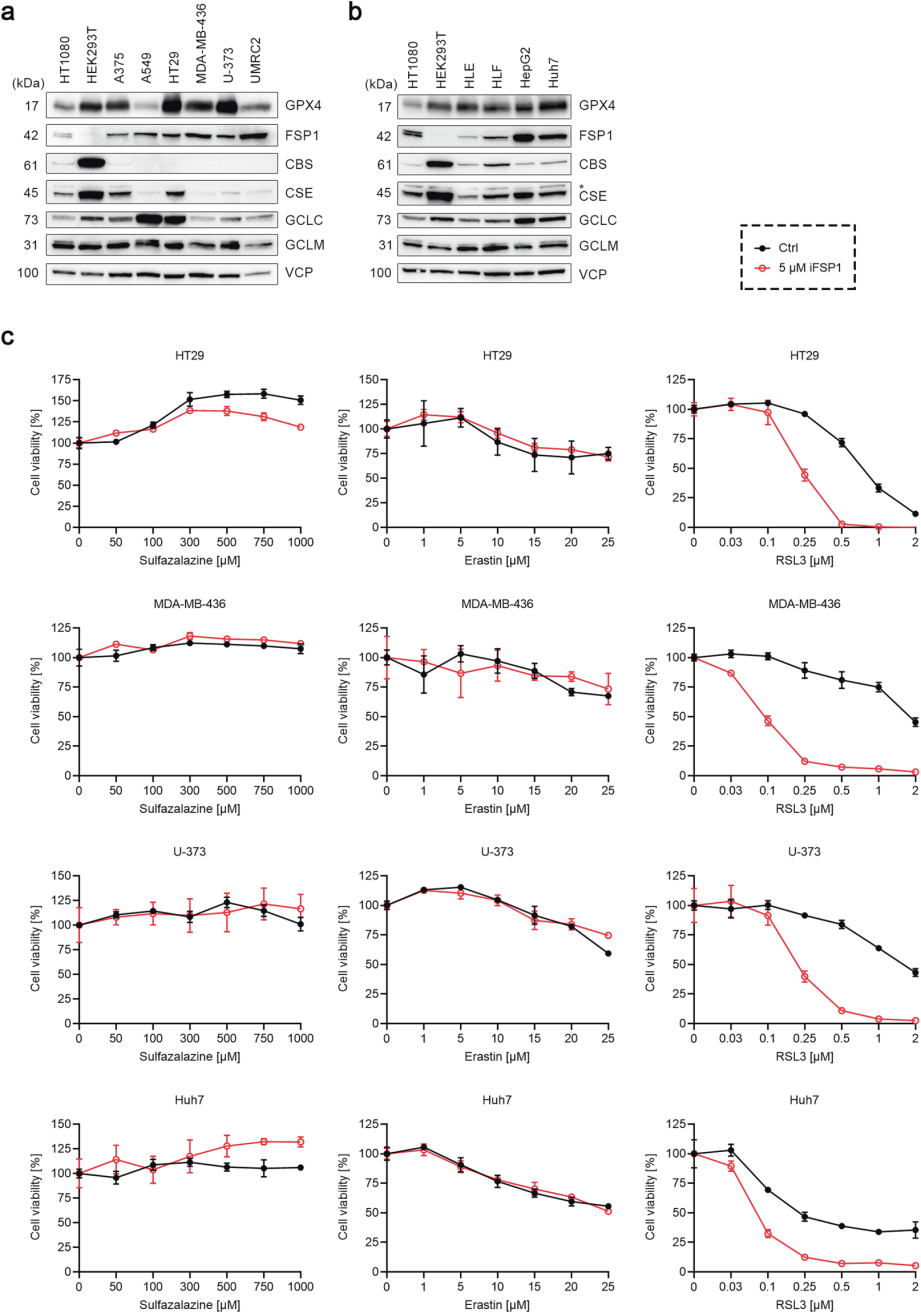
Cells resistant to xCT inhibition are sensitive to co-treatment with RSL3 and iFSP1. **a**, **b** Immunoblots of GPX4, FSP1, cystathionine β-synthase (CBS), cystathionine γ-lyase (CSE), glutamate–cysteine ligase catalytic subunit (GCLC), glutamate–cysteine ligase modifier subunit (GCLM) in HT1080, HEK293T, A375, A549, HT29, MDA-MB-436, U-373, and UMRC2 cells (**a**), and HT1080, HEK293T, HLE, HLF, HepG2, and Huh7 cells (**b**). VCP was used as loading controls. **c** Cell viability of HT29, MDA-MB-436, U-373, and Huh7 cells treated with indicated concentrations of sulfasalazine, erastin, or RSL3 in the absence or presence of 5 μM iFSP1 for 24 h. Data are presented as mean ± s.d. of *n* = 3 wells of a 96-well plate from one representative of three independent experiments.

Since our results showed that some of the cell lines were quite resistant to xCT inhibition, we selected two erastin-resistant cell lines (i.e., Huh7 and MDA-MB-436) to determine whether their intracellular GSH levels were affected by erastin. Compared to WT HT1080 cells, the GSH levels in these two cell lines were equally reduced by erastin, indicating the presence of other mechanisms protecting the erastin-resistant cell lines from erastin-induced GSH loss (Supplementary Fig. S2). We further investigated the protein levels of genes related to cysteine and GSH metabolism (CBS and CSE, two enzymes of the transsulfuration pathway, and GCLC and GCLM, both subunits of γ-GCS) in addition to GPX4 and FSP1, the two key ferroptosis regulatory nodes among different cell lines (Fig. 7a, b). GPX4 expression was relatively constant, except for HT1080 and A549 cells which showed a lower expression. Of note, FSP1 expression was low in HT1080, HLE, and HLF cells, all of which are highly sensitive to erastin-induced ferroptosis. HEK293T cells did not express FSP1, but this cell line displayed a remarkably high expression of CBS and CSE. In addition, CBS and CSE were both detected in HT1080 and hepatoma cell lines. GCLC and GCLM expressions were also similar among different cell lines, except for A549 and HT29 cells which had a relatively high GCLC level.

To determine whether FSP1 plays an essential role in the resistance towards xCT inhibition-dependent cell death, HT29, MDA-MB-436, U-373, and Huh7 cells were treated with increasing concentrations of sulfasalazine or erastin, in the presence or absence of 5 μM iFSP1. As illustrated in Fig. 7c, while iFSP1 failed to sensitize cells to xCT inhibition at least in these four cell lines tested, it significantly increased the cells’ sensitivity toward RSL3 treatment (Fig. 7c, right panels). Hence, these results imply that FSP1 is not the only factor that confers resistance to xCT inhibition-induced cell death, and that some yet-unknown mechanisms might support the function of GPX4 in the absence of xCT.

## Discussion

Unlike other forms of regulated cell death, such as apoptosis and necroptosis, that can be pinpointed by qualified biomarkers (e.g., cleaved caspase-3, phosphorylated MLKL) [25], the validation of ferroptosis so far is largely based on the protective effects of ferroptosis inhibitors, such as RTAs and iron chelators [26]. As such, we used Lip-1 and DFP to determine whether ferroptosis is engaged in the cell death process induced by the compounds used herein. Both Lip-1 and DFP significantly increased the cell viability in HT1080 cells and HLF cells upon erastin or sulfasalazine treatment, demonstrating the effectiveness of these two compounds. Furthermore, among all the cell viability assays we performed, Lip-1 and DFP consistently showed the same rescuing effects, corroborating that both compounds are reliable indicators of ferroptotic cell death. Erastin and sulfasalazine are widely acknowledged system x_c_^−^ inhibitors. Consistent with this, HT1080, HEK293T, and B16F10 cells engineered to overexpress SLC7A11 appeared to be more resistant to these two drugs compared to their respective parental WT cell lines. Furthermore, the fact that β-ME consistently exerted cell-protective effects against the cytotoxicity of erastin and sulfasalazine also indicates that these two compounds are *bona fide* system x_c_^−^ inhibitors [27]. By stark contrast, among all the cell lines included in this study sorafenib conferred equivalent cytotoxic effects which could not be prevented by β-ME, Lip-1, or DFP. *SLC7A11*^*OE*^ HT1080, HEK293T, and B16F10 cells were also equally sensitive to sorafenib compared to their parental WT cells. Based on these findings, we conclude that sorafenib does not qualify as a *bona fide* ferroptosis inducer or even system x_c_^−^ inhibitor.

Lipid peroxidation is considered a hallmark of ferroptosis. Interestingly, we found that sorafenib-induced lipid peroxidation in HT1080 cells could be counteracted by Lip-1. Nevertheless, this data does not necessarily mean that ferroptosis is induced, since the cell death process was not prevented by either Lip-1 or the use of an iron chelator. Indeed, we previously obtained similar results in *SLC7A11*^*KO*^ B16F10 cells, in which case Lip-1 rescued lipid peroxidation but not cell viability [22]. Furthermore, a recent study indicated that strong lipid peroxidation also occurs in non-canonical pyroptosis, albeit it is not involved in pyroptosis execution [28]. As such, ferroptosis should be characterized as a type of cell death that depends on lipid peroxidation for its cytotoxicity. In other words, although lipid peroxidation is a hallmark of ferroptosis, it should not be used as the sole biomarker to validate ferroptosis.

We further found that the combined treatment of RSL3 and iFSP1 readily induced cell death of cells that were resistant to the combined treatment of sulfasalazine/erastin and iFSP1. Consistent with this, *FSP1*^*KO*^ MDA-MB-436 cells were sensitive to RSL3 but not erastin (data not shown). These results imply that inhibition of system x_c_^−^ is not sufficient to impede the function of GPX4 in these cells. Furthermore, among the four cell lines we tested only Huh7 expressed CBS, suggesting that at least the other three cell lines (i.e., HT29, MDA-MB-436, U-373) did not depend on the transsulfuration pathway to provide cysteine [14]. As such, further research is required to investigate how GPX4 functions in this situation.

Another interesting phenomenon we observed is that in some cases (e.g., HEK293T, B16F10, A375 cells) only β-ME but not Lip-1 or DFP increased the cell viability upon erastin or sulfasalazine treatment. Consistent with this, *SLC7A11*^*KO*^ HEK293T and B16F10 cells were not rescued by Lip-1 or DFP, unlike HT1080 cells. Indeed, we previously showed that β-ME withdrawal from *Slc7a11*^*KO*^ B16F10 cells resulted in an impaired cell cycle and endoplasmic reticulum stress, which could not be rescued by Lip-1 or the pan-caspase inhibitor Z-VAD-FMK [22]. These results suggest that for some cell lines cysteine is not only required for GSH synthesis and ferroptosis control but also for some other vital activities.

In conclusion, findings obtained in this study unambiguously demonstrate that sorafenib does not trigger ferroptosis either through inhibition of system x_c_^−^ or some yet-unrecognized ferroptosis-relevant mechanism, unlike erastin and sulfasalazine the *bona fide* system x_c_^−^ inhibitors. In addition, we further found that cell lines may react widely differently towards system x_c_^−^ inhibition: some cell lines seemingly remain unaffected, while others undergo ferroptosis, whereby for the latter a subset undergo cell death which cannot be counteracted by ferroptosis inhibitors. As such, we discourage the usage of sorafenib as a ferroptosis inducer in the future and propose to carefully assess the involvement of system x_c_^−^ in the ferroptotic cell death process.

## Supplementary information

Supplemental material
