# Supplementary material for: Sorafenib fails to trigger ferroptosis across a wide range of cancer cell lines
Source: Cell Death Dis. 2021 Jul 13;12(7):698. doi: 10.1038/s41419-021-03998-w (PMC8277867; doi:10.1038/s41419-021-03998-w)
Supplement: Supplementary file 1 — Supplemental material [file 41419_2021_3998_MOESM1_ESM.pdf]

Figure S1

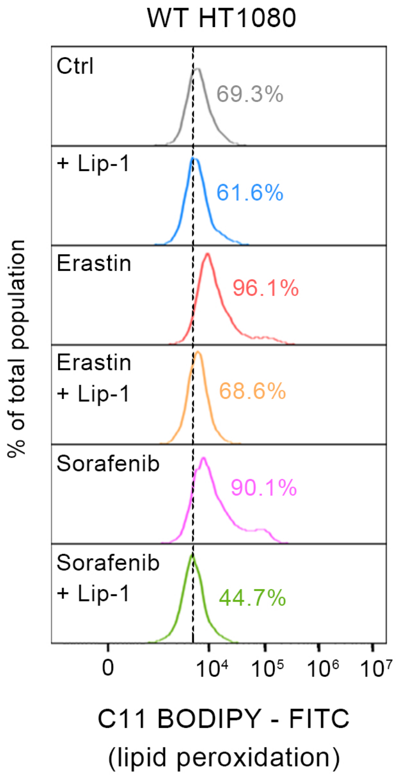

Fig. S1 Sorafenib induces lipid peroxidation in HT1080 cells. Lipid peroxidation assessed by C11 BODIPY using flow cytometry in WT HT1080 cells treated with 10 μM erastin or sorafenib, in the absence or presence of 1 μM liproxstatin-1 (Lip-1) for 4 h. Data are presented from one representative of three independent experiments.

Figure S2

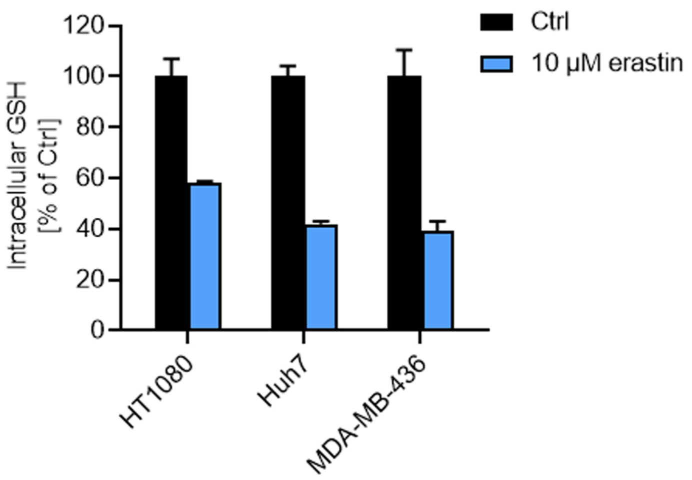

Fig. S2 Erastin induces loss of GSH in erastin-resistant cell lines. Intracellular reduced glutathione (GSH) levels in WT HT1080, Huh7, and MDA-MB-436 cells exposed to 10 µM erastin for 3 h. Data are presented as mean ± s.d. of n = 3 10-cm dishes from one representative of two independent experiments.
